# Implementation Facilitators and Challenges of a Place-Based Intervention to Reduce Health Disparities in Harlem Through Community Activation and Mobilization

**DOI:** 10.3389/fpubh.2022.689942

**Published:** 2022-04-26

**Authors:** Nancy VanDevanter, Lynna Zhong, Rachel Dannefer, Noel Manyindo, Sterling Walker, Victor Otero, Kimberly Smith, Rose Keita, Lorna Thorpe, Elizabeth Drackett, Lois Seidl, La'Shawn Brown-Dudley, Katherine Earle, Nadia Islam

**Affiliations:** ^1^Meyers College of Nursing, College of Global Public Health, New York University, New York, NY, United States; ^2^Department of Population Health, New York University Grossman School of Medicine, New York, NY, United States; ^3^New York City Department of Health and Mental Hygiene, Center for Health Equity, Harlem Neighborhood Health Action Centers, New York, NY, United States; ^4^Community Service Society, New York, NY, United States; ^5^Department of Population Health, School of Medicine, New York University, New York, NY, United States

**Keywords:** structural racism, health equity, public housing, place-based initiative, community mobilization

## Abstract

**Background:**

To address significant health inequities experienced by residents of public housing in East and Central Harlem compared to other New Yorkers, NYC Department of Health and Mental Health (DOHMH) collaborated with community and academic organizations and the New York City Housing Authority to develop a place-based initiative to address chronic diseases in five housing developments, including a community activation and mobilization component led by community health organizers (CHOs).

**Purpose:**

Guided by the Consolidated Framework for Implementation Research (CFIR), we evaluated the initial implementation of the community activation and mobilization component to systematically investigate factors that could influence the successful implementation of the intervention.

**Methods:**

Nineteen in-depth qualitative interviews were conducted with a purposive sample of CHOs, community members and leaders, collaborating agencies and DOHMH staff. Interviews were transcribed verbatim, and themes and codes were developed to identify theoretically important concepts of the CFIR and emergent analytic patterns.

**Results:**

Findings identified important facilitators to implementation: positive community perception of the program, CHO engagement and responsiveness to community needs, CHO norms and values and adaptability of DOHMH and CHOs to community needs. Challenges included the instability of the program in the first year, limited ability to address housing related issues, concerns about long term funding, competing community priorities, low expectations by the community for the program, time and labor intensity to build trust within the community, and the dual roles of CHOs as community advocates and DOHMH employees.

**Conclusions:**

Findings will guide future community activation and mobilization activities. The study demonstrates the value of integrating implementation science and health equity frameworks.

## Background

Community-based interventions have been promoted and implemented by public health experts for many decades with mixed results. A systematic review published in 2003 of 32 community prevention programs found only modest program impacts due to a number of methodological challenges, limitations of the interventions and limitations of theories to guide the interventions (1). As a result, community health researchers, informed by the Ecological Models of Health, shifted focus from individual behaviors to the social environment (2). These models posit that individual behavior interacts with the social environment and are influenced at the inter-personnel, organizational, community and policy levels thus interventions must address multiple levels.

In recent years another challenge, the successful translation of research into practice, has led to a growing interest in the science of implementation. A number of theories and frameworks have emerged from this field to better identify factors that support or inhibit implementation (3). In the last decade, this field has evolved into a greater appreciation of the need for theoretically informed strategies. The Consolidated Framework for Implementation Research (CFIR) (4), a prominent theory in the implementation science field draws on multiple organizational and implementation theories that focus on multiple ecological levels to identify “what works, where and why.” A growing number of implementation studies in the US and Europe have adopted CFIR to evaluate outcomes and to assess constructs that influence implementation (5–8). CFIR has primarily been used in health services research to translate evidence based practices in clinical settings *and has only recently been used in the evaluation of a limited number of community based/community engagement intervention studies* (9). To our knowledge it has not been utilized to assess a multi-component health and capacity-building intervention in a community with significant health disparities.

Decades of research have demonstrated the powerful role social, economic, and political factors play in determining health outcomes. Marginalized racial and ethnic groups experience a disproportionate burden of morbidity and mortality as a result of structural racism, “the totality of ways in which societies foster racial discrimination through mutually reinforcing systems of housing, education, employment, earnings, benefits, credit, media, health care, and criminal justice (10). Due to structural racism and its role in shaping where and how investment or disinvestment occurs, where people are born and live their lives is critical to the opportunities they will have to succeed and thrive (10). The importance of “place” to health outcomes has led to the development of place-based initiatives that focus on improving local conditions to improve health outcomes and health equity (10–14).

The historical context of public housing is critically important to situating the experience of the currently 1.2 million Americans live in public housing (15). The New York City Housing Authority (NYCHA) is the largest housing authority in the country, as well as New York City's largest landlord with responsibility for 5,00,000 residents living in 1,79,000 apartments in 334 developments across the five boroughs of the city (16). Construction of public housing in NYC began in 1934 By 1959, Black and Puerto Rican residents accounted for 57% of families in public housing.

In the mid-1970s, the city was faced with bankruptcy. In the following decades, funding to support social services, including public housing, from city, state and federal agencies has both dramatically declined and been redirected toward other projects in times of budget shortfall (16, 17). There is currently a critical need to improve the “aging infrastructure of the NYCHA properties” without the resources to do so (16, 17). In addition, due to highly segregationist and racially discriminatory housing policies, many NYCHA properties were built in neighborhoods with high proportions of minority residents that were targeted for disinvestment and experienced decades of neglect that leads to poor housing conditions. As a result of these multiple forms of disinvestment, residents of public housing in the US experience many housing related health issues (17).

More robust Interventions to improve the health of people living in communities experiencing significant health inequities are needed. Such interventions have been conducted in multiple US cities to address a wide range of health issues including environmental issues, poor housing, tenants' rights, zoning, neighborhood safety, teen pregnancy and drug use (10–25). Most interventions have been driven by external organizations such as housing and public health agencies with limited input from community members and organizations (16). While research evaluating the health outcomes of public housing interventions is limited, those that have included building the capacity of communities to identify priorities and opportunities have demonstrated positive neighborhood change (10–17).

To address the significant health inequities rooted in racism and experienced by residents in East Harlem public housing compared to other New Yorkers, the New York City Department of Health and Mental Hygiene (DOHMH) worked with local community and academic organizations and NYCHA to establish a place-based initiative to address chronic diseases in five housing developments in 2015 (14). The initiative, known as the Harlem Health Advocacy Program (HHAP), aims to: (1) improve health among residents through health coaching and other place-based wellness activities; (2) support residents to access health and social services to which they are entitled through navigation; (3) build the capacity of residents to seek and create healthy conditions and acceptable services through advocacy to government and other stakeholders; (4) leverage data and partnerships for systems change. Health coaching and other wellness activities are provided by a team of DOHMH community health workers, many of whom are residents of NYCHA. Health and social service navigation is provided in collaboration with Community Service Society (CSS) of NYC, a community-based navigation and advocacy organization.

The HHAP intervention included a community activation and mobilization component. This component consists of a team of five DOHMH community health organizers who interact directly with neighborhood residents to identify issues and advocate for actions to improve neighborhood health. The community health organizers are residents of the developments in which HHAP works. By HHAP's hiring of local residents of public housing, an employment pipeline was created for full-time positions that offer above- industry average pay with benefits. The positions are open to people regardless of education level. This place-based employment model for both community health workers and community health organizers for HHAP was purposely designed to address key social determinants of health for community members (employment, health insurance, income), as well as cultivate interest in careers in health and in public service. It has the added benefit of rooting the program more deeply in the community, increasing the program's credibility and helping to build the trust needed to achieve program effectiveness and sustainability.

The strategy of including community activation and mobilization in this intervention was strongly influenced by the literature on the importance of community organizing and capacity building to address health disparities as the result of structural racism (10–17). This body of work has consistently demonstrated the value of engagement of community members, along with formal and informal organizations to build consensus and strategy to address community priorities and to develop an assets-based approach to community assessment. Building upon this literature, the goals of HHAP's community activation and mobilization component are: (1) to the identify the priorities of residents of NYCHA and develop advocacy strategies to achieve those goals, (2) to increase knowledge about resident's concerns, (3) to strengthen the power of the residents to address health concerns through coalition building, and (4) to mobilize residents to take action to improve health of the community. To achieve these goals, HHAP's community health organizers used a number of strategies including community outreach, organizing community events, meeting with community organizations, assisting community members in solving housing related problems and provided other needed referrals. Details of the intervention design and theoretical framework have been described elsewhere (14).

**Figure 1:**
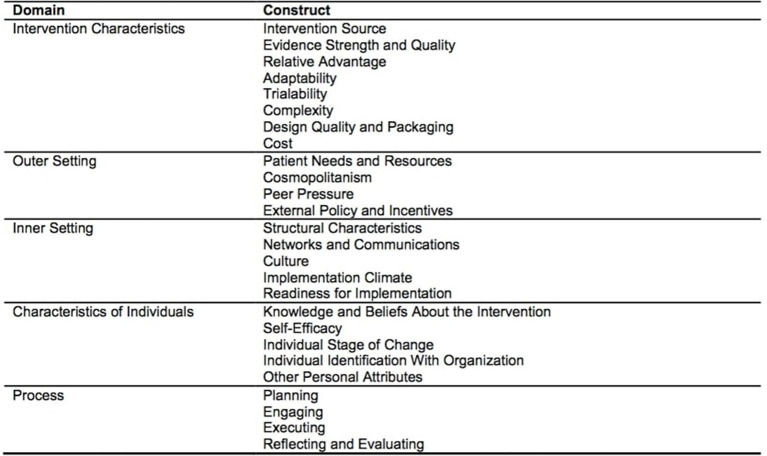
Consolidated Framework for Implementation Research (CFIR) domain and constructs. Source: Damschroder et al. (4).

In this analysis we evaluated the initial implementation of the HHAP community activation and mobilization component. Our objective was to systematically investigate factors that could potentially influence the successful implementation and sustainability of this community engagement model. The Consolidated Framework for Implementation Research (CFIR) (4) guided our baseline evaluation (Figure 1).

## Methods

### Theoretical Framework

The CFIR is an overarching typology for identifying barriers and facilitators influencing implementation of a program or intervention (4). The CFIR is comprised of five domains: (1) Intervention Characteristics (Characteristics of the intervention that may influence success: If interventions are not adapted to the setting they will likely be resisted by the community), (2) Outer Setting (the economic, political, and social context within which an organization resides that may influence success), (3) Inner Setting (internal structural, political, and cultural contexts through which the implementation process will proceed) (4) Characteristics of Individuals (cultural, organizational, professional, and individual mindsets, norms, interests, and affiliations), and (5) Process (the process by which the intervention is implemented). There are 39 constructs associated with specific domains. The domains and constructs represent a synthesis of a range of theories about dissemination, innovation, and implementation.

Over the last decade CFIR has been used to guide assessment of the implementation of a wide variety of studies, settings, and study designs. It has proved to be a valuable tool to guide study design, data collection and analysis and it may be applied at any stage of implementation. In this study, we utilized CFIR to guide the baseline evaluation of HHAP's community activation and mobilization arm to assess the implementation factors that may influence program outcomes that could be modified to increase the potential for program success.

### Sample and Recruitment

Potential participants consisted of a purposive sample of 19 key informants with either direct experience or knowledge of the program during the implementation phase. These individuals were contacted by phone or email by the qualitative researcher (NV) who would conduct the interview and asked if they were willing to participate. The researcher explained that the data would be confidential, and that written consent would not be required to protect their confidentiality. The in-depth, semi-structured interviews were conducted within 6–10 months of initiation of the community activation and mobilization component, which occurred in fall of 2017. *(See interview guide with questions linked to CFIR constructs* Appendix A). To allow for triangulation of community, staff and management perspectives the sample included 4 Community Health Organizers, 4 Department of Health employees associated with HHAP, 4 community residents, 2 representatives from community organizations directly engaged in the community activation and mobilization intervention, 4 community organization leaders and 1 community health worker from the health coaching component of HHAP. The interviews that lasted from 30–60 min were conducted by an experienced qualitative researcher (NV), took place in a private setting of the participants' choosing, were audio-taped with the participants' permission, and transcribed verbatim. The study was approved by the NYU SOM and the NYUDOHMH Institutional Review Boards.

### Data Collection

The interview guide informed by the CFIR explored the participant's background and role in the community activation and mobilization component, perceptions of the design and goals of the program (Characteristics of Individuals and Intervention characteristics), relative priority of the program to the community and the DOHMH (Outer and Inner setting- depending on whether the response is community priority or DOHMH priority), implementation climate readiness (Inner setting and Process) and potential for sustainability. The study was approved by the NYUSOM and DOHMH Institutional Review Boards. To assess the relative priority of the program we asked “What would you say are the most important health issues that need to be addressed in your community?” and “How important are health issues in your community compared to other priorities?” To assess the facilitators and barriers to implementation we asked “What makes it difficult for community organizations like yours to make a positive difference in the community? What would make it easier? ”

### Data Analysis

All interviews were transcribed verbatim and personal identifiers removed. Three members of the research team with qualitative research experience read each of the interviews initially and began to identify potential themes. Subsequently, the team met to refine the initial themes and developed a code book through a process of constant comparison. All transcripts were then coded and entered into Atlas.ti qualitative software. A second researcher coded a 20% subset of the transcripts to establish inter-rater reliability (0.84).

## Results

Findings are organized into facilitators and barriers to implementation of HHAP's community activation and mobilization component utilizing the CFIR framework.

## Implementation Facilitators

Participants identified four CFIR domains and related constructs that have been facilitators to implementation of HHAP's community activation and mobilization component: Outer setting (economic, political and social context within which the organization resides), Inner setting (the structural, political and cultural contexts of the organization), Individual characteristics (characteristics of the individuals involved in the intervention) and Intervention characteristics (the relative advantage of the intervention, adaptability, trialability, complexity, design and quality and cost).

### Outer and Inner Setting: Networking With External (Outer) and Internal (Inner) Organizations

#### Community Engagement and Visibility

HHAP's community health organizers have quickly developed a reputation for being responsive to the needs of community members and have become very visible to the community. Responding to those needs frequently involved collaboration with other community organizations and establishing presence at community events, described below. One community member described her observation of the community health organizers:

“*So, again, they engaged in who they are as a community, allowing people to see them, and they're very visible in the community. Any community board meetings I'm attending, any fairs, PSA 5 meetings, they are there. The resident association meetings, they are there. So, they are very vigilant in the community.”*

A community leader explained how a community health organizer assisted her in meeting a variety of needs for her family:

“*I know they help you with Medicaid and food stamps, even trying to find a decent place and I think that is good. Even as far as finding food for a family at a reasonable price because a lot of supermarkets don't have fresh fruit.”*

A community health organizer described her approach to building connections and visibility in the community:

“*A lot of people I've met over the years I've met through community canvassing, just walk[ing] around on the grounds, going to community centers, playgrounds, through connections from other people, like family or friends who have one of the chronic illnesses that we work with.”*

#### Collaboration With Community Organizations

Similarly, participants noted the collaboration with organizations with a history engagement in policy and advocacy in the target community was essential to establishing community health organizers credibility in engaging in efforts to link community activation with health. One community organization staff described her beliefs about the collaboration:

“*So, there's been a large history at CSS of working on, I guess, you could call them social determinants or working on issues around poverty and health…. the Harlem Health Advocacy Partners and community activation and mobilization component, to me, seems like a response to this moment in time when people are really looking at addressing the disconnect between populations and the care that they're getting. This whole community health work model and movement is really burgeoning right now, really developing, it seems.”*

### Inner Setting: Compatibility

#### Community Health Organizers Live in the Community

The decision to hire community health organizers from the five housing developments was strategic on the part of the NYCDOHMH. Both Community Health Organizers and community members see this as a strong advantage to the community activation and mobilization arm of HHAP.

“*And I love the fact that they are from the community, most of the people that work there. Most of them work there and that's just a phenomenal thing.”*

*Community member*

Participants noted that hiring people from the developments meant that the Community Health Organizers were invested in the work in a unique way.

“*I feel that I care more about residents of our five-targeted developments because I live there. I experience the lifestyle every day. I talk to people in my community every day.”*

*Community health organizer*

“*I think it helps that…we are selecting people who live here because there's that personal investment….This is my building, this is my neighborhood, this is my development*.

*Community health worker*

“*These individuals not only do their nine to five with HHAP, but also have to deal with the truth and realization that there is something that's going on and there is something that I, in my role, should be able to do about that. So I think being able to organize on that level, it's smart that they are from the developments and they have a vested interest in what happens.”*

*DOHMH leadership*

Relatedly, CHOs were also able to bring special expertise through their lived experience as residents of the housing developments to their role with HHAP:

“*It's very important because they know firsthand what's the conditions and what needs to be done.”*

*Community member*

“*Because some of them live in housing, themselves, so they know how to navigate the waters and they can tell the participants how to do that as well.”*

*Community member*

### Characteristics of Individuals: Personal Attributes

#### Norms and Values of Community Health Organizers

Personal characteristics of the community health organizers were described by several participants as important to the successful implementation of community activation and mobilization program. As illustrated by the statements below, community health organizers value working in their communities, and care deeply about health inequities.

“*I like that I work in my community and that it's for the benefit of people in my community that the program is aimed at addressing health inequities and people that are, you know, like dying from unnecessary things that don't have to – that they shouldn't be dying from. You know, like it's 2017 and diabetes is not a death sentence and all this co-morbidity and things, you know, and it's not – so, like the work that I'm doing is able to address that and then I'm working toward that.”*

*Community health organizer*

“*What it comes down to is just people being tolerant and just being aware that everyone comes from a different walk of life but we are all human, and that we are here to do the same work...that we want to serve the community.”*

*Community health worker*

“*But there's a presence in the neighborhood of people who care, people who are vested; who are present, who are present and who are informed, that can be a source, an asset, to promote better health. That's a second benefit: just the mere presence of the team also creates a growing atmosphere of health.”*

*DOHMH leadership*

#### Community Health Organizers Responsiveness to Community Needs

From the perspectives of the community members and leaders, community health organizers have met the needs of the community by providing information and assistance on multiple issues and helping them to navigate systems. One community member described this responsiveness:

“*And so far, like I said, the services that they do provide have been very needed, helpful, and they're wonderful people. I can call them. Actually, Mr. S. has provided me – I needed some material for one of the events that I had. The children have a consent form from their parents and things like that. He sat down with me, and we spoke about it, and he draft[ed] up one, so we had consent forms to go on different trips that we were hosting. So, he provided those services to my group.”*

A community health organizer described the program philosophy:

“*We are not pushing our agenda on them, we're making sure that it's led by the residents and it's what they want to do. We're making it clear that we're here to provide technical support and resources not oversight and dictate what their resident groups do*.

### Intervention Characteristics: Adaptability

In the early stages of the implementation of HHAP challenges arose that required adaptation of the intervention by both NYCDOHMH leadership and community health organizers.

#### DOH Leadership Adaptations

For example, the program initially planned to hire 10 community health organizers on a part-time basis. However, based on feedback from HHAP staff who lived in NYCHA, the program instead created 5 full-time positions with benefits, in order to be more in line with the program's commitment to equity. A DOHMH leaders notes:

“*I do think now that we have the community organizers on board even though it wasn't what we (originally) envisioned; I think they're doing a tremendous job…..”*

*DOHMH leadership*

#### Community Health Organizer Adaptations

Similarly, community health organizers found that the ability to be nimble, creative, and flexible in response to residents' requests enhanced residents' trust of the overall community activation and mobilization activities and likelihood of residents participating in efforts. Community health organizers often worked with residents to resolve specific needs during the initial implementation of the program prior to engaging in efforts or discussions to foster mobilization as a way of building trust with community members:

“*And they can come to this advocate and say, “I'm having problems with mold in my apartment.” “I'm having problems with getting these tickets expedited with NYCHA.” “I'm a sick person, and I don't know what to do anymore.” And one of our residents had said, and she has HIV, and she also goes there, and they helped her with some of her housing issues.”*

*Community member*

## Implementation Challenges

Participants identified four CFIR domains that presented challenges to implementation: Outer setting, Inner setting, Characteristics of Individuals, and Implementation process.

### Outer Setting: External Policies and Incentives

#### Historical Context

The community activation and mobilization component experienced challenges to implementation because of historical factors, community factors and systems issues. The program was initially implemented 1–1/2 years prior to the current evaluation. For complex administrative reasons related to the contracting process the initial program was temporarily postponed, reconfigured and re-implemented in the fall of 2016. Because of this change, community members approach the reconfigured program with some skepticism. A community health organizer explained:

“*My participants on my side of town, they love us….I would say they appreciate it but they want to see consistency, persistence.…because the [community activation and mobilization] was activated before....”*

Another participant observed:

“*…they said that they got disappointed because the people who came out in the beginning, when that [community activation and mobilization team] stopped and ceased to exist, they didn't see them anymore. They didn't see the people who started it that came out and offered to build them up and build their community back.”*

*Community health organizer*

#### Perceived Inability for Community Health Organizers to Directly Impact Larger Housing/Environmental Issues

Community health organizers described some frustration with their inability to impact larger housing related issues for the intervention communities as well as the communities' frustration. In particular, community health organizers noted that though they have had traction in working with individual residents on particular issues, systemic housing deficiencies were challenging to address.

“*And there are a lot of things we don't have the power to resolve for them there. And also, there is just not the capital from within NYCHA, to my understanding, to really create resolution for people around their housing issues, and so that is the big challenge that we encounter.”*

One community health organizer described her perspective on how community members perceive their situation:

“*People are turned off by, yeah; feeling like, ‘my asthma is not a big deal. What about my housing situation?'*

#### Concerns About Long Term Program Funding Support

One community member described some of the confusion of the community:

“*I have no knowledge of future funding.”*

Many HHAP staff expressed concerns consistent with the following statement by a participant:

“*I would love to think there is an institutional commitment to continuing it, and to expanding it, and to improving it, really. That would be exciting. I don't know where that would come from, where that kind of funding would come from on a longer term.”*

### Outer Setting: Peer Pressure

#### Competing Organizations in the Environment

One DOHMH participant explained the challenges of organizations working in the area:

“*So, I think one of the challenges might be navigating that very diverse array of players in these spaces. And sometimes perhaps coming with a different history in the way that they were formed and what issues they were set up to tackle, and how, maybe, they evolve overtime or not evolved over time. And understanding all those issues and navigating the possible politics that comes with work like this.”*

An example, a community activation staff member described the challenge of working with elected members of the tenants' association in the housing communities:

“*The TA (Tenants' Association) Presidents can okay for you to use space and things, so if they're not the easiest to work with then that may not happen.”*

### Outer Setting: Resident Needs and Resources

#### Urgency of Housing and Socio-Economic Conditions

Due to the multiple challenges community members experience, community health organizers noted that residents were often so focused on more immediate challenges that their personal health was a less urgent issue. One community health organizer described what he believes is the community perspective:

“*I don't feel like there is a sense of urgency when people hear about the program (HHAP). I don't feel like people feel like they need to manage their health.…they look at our service as a supplementary.…, it's an add on.”*

Another CHO participant explained the priorities of community members:

“*....health is so far out of touch for us - and I say us because I do live here - it's the little things that we deal with on a daily basis, if we wake up, we give thanks to God, and his glory, and we take our medicine and keep on trucking.”*

Competing priorities identified by community and staff participants included neighborhood safety, housing conditions, and food insecurity. One participant describes this challenge below:

“*When you talk about healthy eating as it relates to diabetes, and you have a resident that hasn't had a stove for months in their apartment, how are we beginning to have that conversation with them? ”*

#### Hesitancy to Become Involved in Community Activation and Mobilization

Community health organizer advocates observed that some residents seemed hesitant to engage in advocacy through HHAP:

“*They want to come. They say they'll come, then they don't. They get involved but then they disengage.”*

Other community health organizers described similar experiences:

“*I thought I had a group of people together who seem pretty interested and I was emailing them and trying to reach out to them and they really weren't getting back to me and one I even screened for the program and she didn't follow up for whatever reason.”*

Another community health organizer describes community member's reluctance to engage in advocacy efforts as a challenge to community activation and mobilization:

“*The resident's hesitancy to help join the program and to come out to things, to be part of it. That is what makes it the hardest I feel like.”*

### Inner Setting: Network and Communication

#### Need to Expand Marketing

The outer setting challenge of community members' lack of awareness of community programs and resources and hesitancy to get involved in community activation and mobilization efforts was amplified by related inner setting communication challenges. Specifically, many participants discussed the need for greater visibility for this part of HHAP. One community health organizer suggested an approach to achieve this goal:

“*If we were able to have an Instagram page and Facebook page and invite everyone within these two Zip codes and do a targeted marketing strategy, I think if we were able to do that, then we would have a lot more people engaged in the program, not just on social media but they would be aware of different events and programs that we have going on so they'd be, they would have more access to what we are doing vs. word-of-mouth and phone calls.”*

One community leader stated:

“*But there needs to be more light shined on it. There definitely does because they are doing good things. The people are phenomenal.”*

### Inner Setting: Readiness for Implementation

#### Time and Effort to Build Trust With Community

The community health organizers acknowledged the challenge of building relationships with the community impacted the readiness for implementation, and that in fact the engagement process itself was time-intensive:

“*And it has taken up a lot of labor, time, organizing power, strategizing and sort of all under the radar because we have to build this project in the community where we really had to build it from the ground up.”*

The legacy of disinvestment and lack of true support from government institutions contributed to challenges in building trust:

“*We have, certainly experienced pushback because there's just the sense when you see, historically, that your population that is just not valued by political figures, I think there's a tendency to distrust the idea that you could actually change that perspective. And so, working within the government of health, even though people like the program, and they like working with us, and they like to engage, there is still like an, oh, yeah, well, you're just gonna put this back on us to deal with and to solve. And you're not gonna solve anything for us.”* Community Health Organizer/.

Additionally, initially being personally unknown to residents presented a challenge in building trust with community members:

“Before I was really connected with my community, they looked at me as an outsider, as an entity, rather than a tenant of [public housing complex]..” Community Health Organizer

One community leader confirmed this challenge:

“*And it's not because they're not making efforts to go out into the community and reach out to the community. It's more of the community just accepting change.”*

### Characteristics of the Individuals: Individual Identification With the Organization

#### Dual Role as Community Advocates and Employees of DOHMH

Program staff participants described some of their own challenges, including the fact that Health Department employees are restricted from engaging in political advocacy within their work roles. One described the “split” between the two roles, being a community member and being an employee of the program, this way:

“*…We can't really protest. We can't do certain things because we ARE affiliated with DOHMH, but it's hard because at the same time since we are putting on this activist hat and are natural residents, you kind of don't want to stop yourself from being revolutionary in some ways. Or sometimes it feels like a little bit of a problem to feel like you have to choose between, you know, being a resident and being an employee here, even though you are working with residents.”*

### Implementation Process: Engaging

#### Challenges to Evaluating Impact of Community Activation and Mobilization Efforts

One DOHMH leader explained the challenge:

“*So if you're solely looking at, let's say, A1Cs as a sole way to turning the program into a success, then you're gonna miss out the other key pieces and components of the program. You're going to look at it very narrowly and more on an individual basis and not so much on a population basis. And to understand that there are policies that have to be enacted in order for the population at large to benefit from health outcomes, for their health outcomes to improve. And then there's this idea of replication, like how do we prove the program's success? How do we get folks to understand the different pieces and how intricate they work together? And how do we allow something like this to be replicated throughout the city, possibly?”*

## Discussion

Our findings contribute the literature on the impact of community-based health interventions aimed at increasing community activation and mobilization. In particular, this study assessed barriers and facilitators to a place-based community intervention that aimed to increase community activation and mobilization in order to reduce health disparities in public housing. The majority of community-based intervention studies have not examined factors associated with program implementation. To our knowledge, our study represents one of the first the first to use the CFIR framework to explore the implementation of community organizing efforts. By grounding our analysis in the CFIR framework, our study makes a unique contribution to the field of place-based interventions to reduce health disparities. The study identified important facilitators to implementation of the community activation and mobilization component of HHAP to meet the principal program aim of reducing health disparities due to historic structural racism.

Our study builds on past implementation literature by using CFIR constructs to identify potential short or long-term barriers to implementation and sustainability that need to be addressed as the program continues to grow (4). Similar to other implementation studies, this study found that CFIR provided a valuable tool for assessing domains and constructs important to successful intervention implementation (8).

Findings from the study provide guidance for adaptations that could increase the potential for successful study outcomes. Outer setting issues like the history of discontinuing the original program will, in some cases, require discussion with community members, but have become less important as the program continues to grow and demonstrate success. Community health organizers have addressed the current challenge of resident concerns about housing conditions through extensive efforts to provide information and support for navigating NYCHA, thereby establishing trust with residents. However, our findings point to the need for the community activation and mobilization program to support residents in developing strategic plans to address systemic changes needed to improve housing conditions. Follow-up assessment of the community activation and mobilization component can assess the extent to which community health organizers have been successful in building a base of residents to engage in advocacy efforts as well as growing partnerships with external organizations that can facilitate advocacy. It can also assess the extent to which the Health Department is able to play a role in larger policy and systemic changes around housing. This is related to the other identified Outer Setting issue of competition among various community organizations, which can be addressed by continued efforts to enhance communication and further engagement of community members with ties to those organizations. While the programs long-term funding is now secure, as the program has been baselined in to the DOHMH budget at the city level, a clear statement to community members and other stakeholders about the long-term funding commitment could address community members' concerns.

Inner setting issues identified primarily can be addressed by supporting the community health organizers' efforts to recruit, understanding the challenges they face and the time and intensity of recruitment. The continued success of HHAP's community activation and mobilization component will enhance the community's knowledge about available services and potentially how to integrate health with other priority issues. A formal multi-pronged marketing plan for the community activation and mobilization component could reduce the time and intensity for recruitment. Notwithstanding the built-in limitations around city government staff engaging in advocacy, better clarification of the parameters of the dual role the community health organizers play as members of the community and as DOHMH employees would reduce some of the stress they experience.

As a qualitative study there were several important limitations. First, the data was collected only from people directly involved in the implementation of the intervention. Other stakeholders not directly involved were not included in the sample. We did interview recipients of the intervention but only those who were directly involved in receiving the intervention in its current mode. We were limited in the use of the CFIR to the stage of the intervention implementation which was 6–12 months so we have no information about the long term and we did not focus on the preliminary Process constructs such as planning, engagement, executing, reflecting and evaluating as many of the staff and community member involved in the initiation of the programs 2 years previously were no longer available. We were also limited to our ability to assess the impact of individual characteristics on the implementation.

Despite these limitations, our study makes an important contribution to the field of implementation science by utilizing the CFIR framework to assess the implementation of a place-based initiative with a broad goal of empowering community residents to advocate for change to improve health outcomes. As our nation continues to grapple with the persistent and insidious role of structural factors in perpetuating health inequities, understanding successful mechanisms to build community capacity and power are critical.

## Data Availability Statement

The raw data supporting the conclusions of this article will be made available by the authors, without undue reservation.

## Ethics Statement

The studies involving human participants were reviewed and approved by the New York University (NYU) Grossman School of Medicine Institutional Review Board. Written informed consent for participation was not required for this study in accordance with the national legislation and the institutional requirements.

## Author Contributions

All authors have contributed substantially to the article and have approved the version being submitted.

## Funding

This study was funded by New York City Department of Health and Mental Hygiene. NI's time is partially supported by the National Institute on Minority Health and Health Disparities Grant U54MD000538, Centers for Disease Control and Prevention Grant U48DP001904, and National Institutes of Health National Center for Advancing Translational Science Grant UL1TR001445.

## Conflict of Interest

KE was employed by Community Service Society. The remaining authors declare that the research was conducted in the absence of any commercial or financial relationships that could be construed as a potential conflict of interest.

## Publisher's Note

All claims expressed in this article are solely those of the authors and do not necessarily represent those of their affiliated organizations, or those of the publisher, the editors and the reviewers. Any product that may be evaluated in this article, or claim that may be made by its manufacturer, is not guaranteed or endorsed by the publisher.
