# Supplementary material for: Implementation Facilitators and Challenges of a Place-Based Intervention to Reduce Health Disparities in Harlem Through Community Activation and Mobilization
Source: Front Public Health. 2022 Apr 26;10:689942. doi: 10.3389/fpubh.2022.689942 (PMC9090448; doi:10.3389/fpubh.2022.689942)
Supplement: Supplementary file 1 [file Data_Sheet_1.pdf]

Appendix A

Key Informant Interview Guide

Community Activation Teams

Thank you very much for being willing to talk to me today about your experience with the Community Activation Team or CAT. We are asking people like you to tell us about your experience with CAT, in what ways you think it could help the community and what is needed for it to succeed. If you agree, I would like to record our discussion to help me remember your recommendations. I will destroy the tape once I have written a summary of what we discussed. Please feel free to say whatever you want to about the program. Whatever you say will be totally confidential and your name will never be used in any reports about the program. You may refuse to answer any question you do not wish to answer and you may stop the interview at any time. Do you have any questions before we begin?

1. How would you describe the main goals of the CAT component of HHAP?

   Probe: What are the main problems that the CAT tries to address?

2. How important do you think the problems are to the community that the CAT is designed to address compared to all of the other problems affecting the community? **(Outer Setting-Patient Needs &Resources, Intervention Characteristics-Relative Advantage, Inner Setting-Relative Priority)**

3. How effective do you think the CAT strategies are to addressing the problems of the community? **(Intervention Characteristics-ALL constructs)**

   Probe: What is needed for them to be successful? (skilled leaders, action plan, resources, commitment of community members, commitment of government agencies to collaborate with them?)

4. What improvements do you think have been made in the community to meet the needs identified : **(Intervention Characteristics-Relative Advantage, Adaptability, Design Quality & Packaging)**

   Probe: By the HHAP program? The community at large?

5. What are the major challenges to reaching the CAT goals? What are the major facilitators to achieving the CAT goals?

6. What kind of support is available to CAT community organizers to carry out their responsibilities? **(Inner Setting-Leadership Engagement)**

Probe: What additional supervision, training or resources would enhance the CAT role in the intervention?

6.What kind of supports like online resources, health education materials and social media access would enhance the CAT program?

Probe: What would make it more successful?

7.What kind of life experience or training did you have when you took this position?

***Probe: Are there additional skills you would like to acquire as a result of the program that you think would make it more effective? (*Intervention Characteristics, Design, Quality and &Packaging)**

8.How confident are you that you can make a difference in achieving the short and long term goals of CAT? (**Characteristics of Individuals- Self- Efficacy, Collective Efficacy)**

9.What would you like to see CAT and HHAP accomplish this next year?

Probe: What might be some challenges to accomplishing it? What would help?

10.What resources would be needed for the program to continue?
(Outer Setting-Patient Needs and Resources)

11. What kind of local, state or national policies might influence the decision to continue the program?
(Outer Setting-External Policy & Incentives; Inner Setting- Implementation Climate- Relative Priority)
11.How confident are you that CAT will make a positive difference in this community in the future?

Probe: Why do you think that it will be successful or not? (Challenges and facilitators)

Thank you very much for sharing your thoughts about CATS and HHAP today. Are there any additional thoughts or questions that you have for me?
